# Effect of case management on neonatal mortality due to sepsis and pneumonia

**DOI:** 10.1186/1471-2458-11-S3-S13

**Published:** 2011-04-13

**Authors:** Anita K M Zaidi, Hammad A Ganatra, Sana Syed, Simon Cousens, Anne CC Lee, Robert Black, Zulfiqar A Bhutta, Joy E Lawn

**Affiliations:** 1 Department of Paediatrics and Child Health, the Aga Khan University, Karachi, Pakistan; 2 London School of Tropical Medicine and Hygiene, London, UK; 3 Johns Hopkins Bloomberg School of Public Health, International Health, Baltimore MD, USA; 4 Saving Newborn Lives/Save the Children, Cape Town, South Africa

## Abstract

**Background:**

Each year almost one million newborns die from infections, mostly in low-income countries. Timely case management would save many lives but the relative mortality effect of varying strategies is unknown. We have estimated the effect of providing oral, or injectable antibiotics at home or in first-level facilities, and of in-patient hospital care on neonatal mortality from pneumonia and sepsis for use in the Lives Saved Tool (LiST).

**Methods:**

We conducted systematic searches of multiple databases to identify relevant studies with mortality data. Standardized abstraction tables were used and study quality assessed by adapted GRADE criteria. Meta-analyses were undertaken where appropriate. For interventions with biological plausibility but low quality evidence, a Delphi process was undertaken to estimate effectiveness.

**Results:**

Searches of 2876 titles identified 7 studies. Among these, 4 evaluated oral antibiotics for neonatal pneumonia in non-randomised, concurrently controlled designs. Meta-analysis suggested reductions in all-cause neonatal mortality (RR 0.75 95% CI 0.64- 0.89; 4 studies) and neonatal pneumonia-specific mortality (RR 0.58 95% CI 0.41- 0.82; 3 studies). Two studies (1 RCT, 1 observational study), evaluated community-based neonatal care packages including injectable antibiotics and reported mortality reductions of 44% (RR= 0.56, 95% CI 0.41-0.77) and 34% (RR =0.66, 95% CI 0.47-0.93), but the interpretation of these results is complicated by co-interventions. A third, clinic-based, study reported a case-fatality ratio of 3.3% among neonates treated with injectable antibiotics as outpatients. No studies were identified evaluating injectable antibiotics alone for neonatal pneumonia. Delphi consensus (median from 20 respondents) effects on sepsis-specific mortality were 30% reduction for oral antibiotics, 65% for injectable antibiotics and 75% for injectable antibiotics on pneumonia-specific mortality. No trials were identified assessing effect of hospital management for neonatal infections and Delphi consensus suggested 80%, and 90% reductions for sepsis and pneumonia-specific mortality respectively.

**Conclusion:**

Oral antibiotics administered in the community are effective for neonatal pneumonia mortality reduction based on a meta-analysis, but expert opinion suggests much higher impact from injectable antibiotics in the community or primary care level and even higher for facility-based care. Despite feasibility and low cost, these interventions are not widely available in many low income countries.

**Funding:**

This work was supported by the Bill & Melinda Gates Foundation through a grant to the US Fund for UNICEF, and to Saving Newborn Lives Save the Children, through Save the Children US.

## Background

Deaths occurring in the neonatal period each year account for 41% (3.6 million) of all deaths in children under 5 years [1]. The majority of these deaths occur in low income countries and almost 1 million of these deaths are attributable to infectious causes including neonatal sepsis, meningitis and pneumonia [1]. These deaths occur because of lack of preventive care (clean birth care, breastfeeding) and appropriate case management [2]. Delays in treating neonatal infections of even a few hours may be fatal. Delays in illness recognition and care seeking, a dearth of primary health care providers, and limited access to facility care contribute to these deaths [3]. Recent trials have demonstrated the effect of community-based packages for prevention and treatment of neonatal bacterial infections, with the potential to save many lives [4,5].

Therapy with appropriate antibiotics and supportive management in neonatal nurseries is the cornerstone of management of neonatal sepsis and pneumonia, with strong biological plausibility that such therapy saves lives. Yet the quality of evidence is understandably affected by the ethical impossibility of undertaking randomized trials of antibiotic management compared with no antibiotic management. Nevertheless, given the limited access to care for sick neonates in low income countries, it is important to assess the potential mortality effect of oral antibiotics and injectable antibiotics delivered in domiciliary or primary care settings. Case management for hospitalized neonates is more expensive, but to guide policy and program investments we also need to know how much more effective it is compared to care delivered at home or in primary care settings.

The objective of this review is to provide estimates of the effectiveness of three interventions in preventing neonatal deaths from severe infection: (i) case management with oral antibiotic therapy alone for pneumonia and sepsis; (ii) case management with injectable antibiotics (± oral antibiotics) as an outpatient or at home for neonatal sepsis /meningitis and pneumonia; and (iii) hospital-based case management, including injectable antibiotics, intravenous fluids, oxygen therapy, second line injectable antibiotics if needed, and other supportive therapy (Table 1). These mortality effect estimates are used in the Lives Saved Tool (LiST) software, a user-friendly tool that estimates the number of lives saved by scaling up key interventions and helps in child survival planning in low income countries [6,7].

**Table 1:** Definitions of interventions reviewed

Oral antibiotic therapy alone • Administration of oral antibiotics in the community for neonatal sepsis, meningitis, or pneumonia
**Injection therapy alone** • Administration of intramuscular antibiotics, at home or in first-level facilities, for neonatal sepsis, meningitis, or pneumonia

**Hospital-based management as an inpatient with supportive care** • Administration of intravenous antibiotics ○ Wider choice of antibiotics including broad spectrum antibiotics ○ Option of using frequent/higher dosage if needed to maintain high blood antibiotic levels or coverage for meningitis, ○ Access to second-line antibiotic therapy for neonates with treatment failure on first line antibiotics • Intravenous access and administration of intravenous fluids if needed • Oxygen supplementation if required • Access to appropriate diagnostic procedures, such as monitoring of pulse, blood pressure, and oximetry reading, as well as monitoring/correction of hypoglycemia if required

## Methods

### Searches

**Figure 1:**
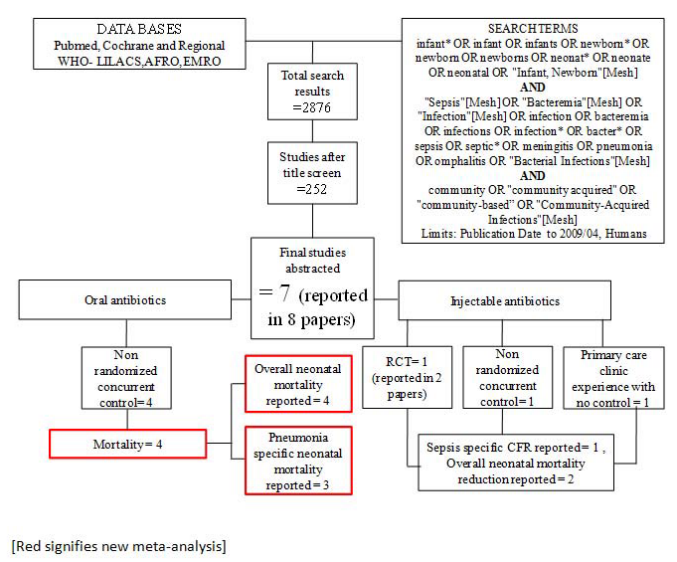
Searches and screening for community based management of sepsis and pneumonia.

**Figure 2:**
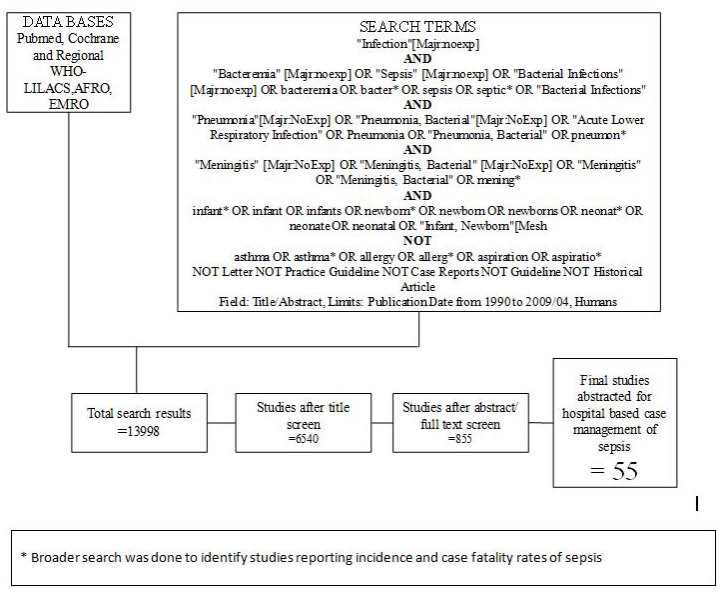
Searches and screening for hospital management of sepsis

**Figure 3:**
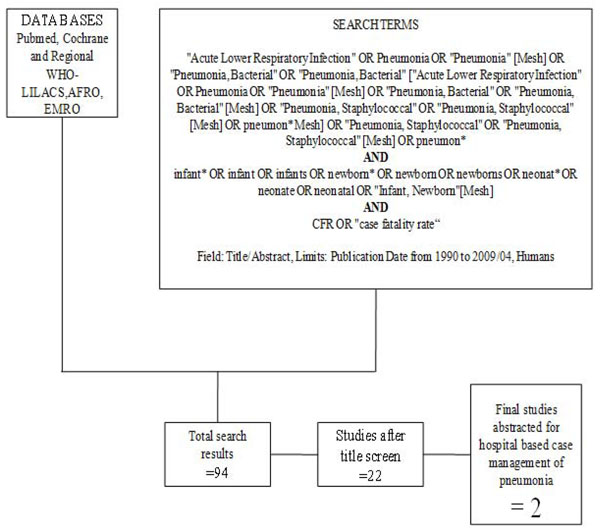
Searches and screening for hospital management of pneumonia.

We searched all published literature as per CHERG systematic review guidelines[7]. Databases searched were PubMed, Cochrane Libraries and WHO regional databases from 1990 until April 2009 and included publications in any language (Figure 1). Search terms included various combinations of: sepsis, meningitis and pneumonia. For sepsis and pneumonia management at a hospital level we conducted two parallel searches (Figures 2 and 3). These were broader as we also wanted to identify studies reporting incidence and case fatality ratios (CFR) for a related study on global burden of neonatal sepsis. Titles and abstracts were reviewed and studies were included if data on one of the following outcomes was provided: all-cause mortality, sepsis/meningitis/pneumonia mortality and/or CFR. Furthermore, extensive efforts were made to contact investigators and program managers for unpublished data.

### Inclusion/exclusion criteria, abstraction

We reviewed all available observational studies, randomized controlled trials, systematic reviews, and meta-analyses, which included neonates and principally involved the management of serious neonatal infections. The search was limited to “humans”. We examined studies published from 1990 until April 2009.

We included randomized controlled trials, studies with concurrent controls, and observational studies with no control group if mortality outcomes were reported. All studies meeting final inclusion criteria were double data abstracted into a standardized form. We abstracted key variables with regard to the study identifiers and context, study design and limitations, intervention specifics, and mortality outcomes. We assessed the quality of each of these studies using a standard table employing an adapted version of GRADE[8] developed by the Child Health Epidemiology Reference Group (CHERG) [7]. For studies which reported mortality outcomes that were not neonatal specific, we contacted the authors to request the neonatal-specific data. All studies that were coded are included in Additional File 1.

### Case definition of sepsis

During our review of selected studies we were unable to find a standard definition for clinical neonatal sepsis or pneumonia (Table 2). Each study used different criteria although most are a variation on WHO IMCI approach. We therefore decided to accept authors’ definitions of sepsis and pneumonia, recognizing that these non-specific definitions lower mortality outcome estimates as many “non-sepsis” cases are included in an effort to maximize sensitivity.

**Table 2:** Varying definitions of neonatal sepsis used by investigators and clinicians

Common signs of neonatal sepsis:	
• Lethargy or irritability • Poor feeding • Vomiting • Jaundice	• Respiratory distress • Apnoea • Fever or hypothermia
**Definition of neonatal sepsis used by Bang et al (1999)**[9] Presence of two or more of the following signs:	
• Weak or absent cry • Weak or reduced suckling • Drowsy or unconscious baby • Temperature more than 37.2°C or less than 35°C	• Diarrhoea or persistent vomiting or abdominal distension • Grunting or severe chest indrawing • Respiratory rate of 60 or more • Pus in skin or umbilicus

**Definition of neonatal sepsis used by Baqui et al (2008)**[12] Presence of one or more of the following signs:	
• Convulsions • Unconsciousness • Fever ≥ 38.3°C • Breathing ≥ 60 per minute	• Body Temperature ≤ 35.3°C • Many or severe skin pustules or blisters on single large area, or pus or redness with swelling Severe chest indrawing

**Definition of sepsis by Young Infant Clinical Signs Study Group (2008)**[84] Presence of one or more of the following signs:	
• Difficulty feeding • Convulsions • Movement only when stimulated • Respiratory rate of 60 or more	• Severe chest indrawing • Temperature ≥ 37.5°C • Temperature ≤ 35.5°C

### Analyses and summary measures

All studies reporting mortality data for pneumonia and sepsis management, in community and hospital settings, were summarized according to the overall quality of evidence for each outcome and each data input type using an adapted version of the GRADE 21 protocol table [7]. When appropriate, we conducted meta-analyses to obtain pooled estimates of the risk ratios, using either the Mantel-Haenzsel or, when there was evidence of heterogeneity, the DerSimonian-Laird random effects estimator. 95% confidence intervals (CI) were also calculated. Statistical analyses were performed using STATA 10.0 (http://www.stata.com).

### Delphi Process for Establishing Expert Consensus

For intervention-outcome combinations for which we did not identify moderate quality evidence, we sought expert consensus via the Delphi method. Individuals invited to participate were experts in newborn health and sepsis representing six WHO regions (South Asia, Africa, Western Europe, Eastern Europe, North America, Australia), and including multiple disciplines - international health, pediatric infectious diseases, clinical neonatology, and general pediatrics. Twenty (of twenty-three experts invited) agreed to participate in the Delphi process. The questionnaire was developed by JL, AZ, SC and SS, and refined after several rounds of pilot testing. The questionnaire was sent by email and included the background and aims of the Delphi and estimates of effect that were available from the literature for different scenarios. The median response and range were determined for each question. Consensus was defined a priori as an interquartile range in responses of not more than 30% for each question. For those estimates not reaching consensus, the plan was for results to be electronically distributed to the panel, virtual discussion allowed, and a second round of email questionnaires sent. However, consensus was achieved after one round of questionnaires and subsequent rounds were not necessary.

## Results

### Studies identified

Our systematic searches for community management of sepsis and pneumonia identified 2876 titles (Figure 1) and after screening of titles, abstracts and relevant full texts, we located 7 studies of interest (reported in 8 papers) [9-16]. We identified 4 non randomised concurrently controlled studies, which evaluated oral antibiotics for pneumonia (Table 5) [10,14-16]. Three of these studies did not report disaggregated neonatal outcomes in the primary papers, but neonatal outcomes were available through abstracted forms from an earlier meta-analysis by Sazawal et al [17]. For management of neonatal sepsis using injectable antibiotics, we located 3 studies (reported in 4 papers) [9,11-13]. There was one observational primary clinic-based study without a control group [13], one RCT [12] and one non-randomised, concurrently controlled study [9]. The fourth paper reported observational data from individual infants evaluated during the RCT mentioned above and was not a separate study [11]. All the studies were from high neonatal mortality regions.

In our search for hospital-based studies of sepsis we found 55 studies from a total pool of 13998 studies which reported sepsis and/or meningitis mortality outcomes (Figure 2) [18-70]. For pneumonia, we found two studies from a total pool of 94 studies (Figure 3) [71,72].

The details of each study and quality assessment using GRADE are summarised in Tables 3, 4, 5, and 6.

**Table 3:** GRADE assessment of studies of effect of case management on cause specific neonatal mortality due to pneumonia

	Quality Assessment					Summary of Findings		
						**No. of Events**		**Effect**

**No. of studies**	**Design**	**Limitations**	**Consistency**	**Generalizability to Population of Interest**: means to the “population”	**Generalizability of the Intervention of interest**	**Intervention**	**Control**	**Relative Risk (95% CI)**

***Mortality Pneumonia – community based oral antibiotic studies***								

4	1 randomized 3 Non randomized - concurrent control	Studies are not randomized, coverage of intervention are estimates, exact data not available, intensity of co-interventions varies between studies	Findings from the 4 studies all show direct mortality reduction benefit, although in 3 of the 4 studies included in the meta analysis, the effect reduction is not significant.	Yes, studies were all done in high neonatal mortality regions.	3 of the 4 studies show direct effect on pneumonia specific mortality. 1 shows effect on overall neonatal mortality	248/ 6542	63/ 4538	*All-cause mortality 0.75 (0.64- 0.89) **Pneumonia Specific 0.58 (0.41- 0.82)

***Mortality Pneumonia - community based injectable antibiotic studies***								

No studies identified								

***Mortality Pneumonia - hospital-based case management***								

2	Both observational study design	Not trials	CFR: 14.4% (28/195) and 30.8% (8/26)	Both studies from low income South Asian countries.	The study reporting higher CFR had high proportion (60%) of LBW babies.	N/A	N/A	N/A

**Table 4:** GRADE assessment of studies of case management on cause specific neonatal mortality due to neonatal sepsis

	Quality Assessment					Summary of Findings		
						**No. of Events**		**Effect**

**No. of studies**	**Design**	**Limitations**	**Consistency**	**Generalizability to Population of Interest**	**Generalizability of the Intervention of interest**	**Intervention**	**Control**	**Relative Risk (95% CI)**

***Mortality Sepsis – community based oral antibiotic studies***								

No studies identified								

***Mortality Sepsis – community based injectable antibiotic studies***								

2	Observational	1 study has no control group	Yes: both show low CFRs (3.3%, 4.4%)	Yes, both studies were done in high neonatal mortality regions.	Direct	133/2211	N/A	N/A

1	Non randomized - concurrent control trial	Change in sepsis specific mortality rate in intervention and control areas is not given	The results of this study were consistent with the RCT	Yes, study was done in a high neonatal mortality region.	Indirect	54/1783	113/2048	0.56 (0.41-0.77)

1	RCT	Sepsis specific reduction in mortality not given	Reported similar results as study above	Yes, study was done in a high neonatal mortality region.	Indirect	82/2812	125/2872	0.22 (0.07-0.71) CFR=4.4%

***Mortality Sepsis/Meningitis - case management in hospitals***								

55	All observational study designs	All observational with varied study setting, from high-income to low-income countries. In low-income countries self-selecting populations because most births happen at community level.	CFR range from 67 to 6.7%	*NMR LEVEL5= 5 studies NMR LEVEL4=17 studies NMR LEVEL 3= 5 studies NMR LEVEL2=5 studies NMR LEVEL1=22 studies Multi country=1	In countries with high skilled attendance hospital data generalizable to all population. But in low-income countries, hospital data not given as most births at home	N/A	N/A	N/A

*NMR LEVELs (1=NMR <5 per 1000 live births, 2=NMR 6 to 15 per 100 live births, 3= NMR 15 to 30 per 100 live births, 4=NMR 31-45 per 100 live births 5=NMR >45 per 100 live births

**Table 5:** Summary of community-based studies for case management with oral antibiotics for and effect on cause specific neonatal mortality due to pneumonia

Ref and year	Country	Setting	Study design	Therapy given	Other interventions in package	Coverage of antibiotic case management (% of those who need it)	Intervention group (N/D)	Control group (N/D)	Effect size RR (95 % CI)	
									
									RR of Sepsis specific NMR	RR of Pneumonia specific NMR
**Pandey 1991**[16]	Nepal	Rural	Non randomized -concurrent control	Cotrimoxazole 4 mg/kg BD for 5 days. Chloramphenicol if no improvement	Maternal education, and 15% measles immunization coverage of children	<40-70% (estimates as per study PIs)	81/681	16/681	0.85 (0.65-1.12)	0.89 (0.46-1.72)
**Mtango 1986**[15]	Tanzania	Rural	Non randomized -concurrent control	Cotrimoxazole PO	Health education to mothers about symptoms & signs of ARI and referring severe cases to the next higher level of care.	<40-70% (estimates as per study PIs)	37/1638	7/1638	0.70 (0.47-1.07)	0.44 (0.18-1.07)
**Khan 1990**[14]	Pakistan	Rural	Non randomized -concurrent control	CotrimoxazolePO	Qualified nurses monitored and supervised CHW activities and with assistance of the CHWs, conducted frequent, informal, interactive health education programs	<40-70% (estimates as per study PIs)	26/2690	9/686	0.74 (0.35 - 1.57)	Did not report pneumonia specific mortality
**Bang 1990**[10]	India	Rural	Non randomized -concurrent control	Cotrimoxazole 2.5 ml twice daily for 7 days	Mass health education about childhood pneumonia	76% (for children <5)	104/1533	31/1533	0.70 (0.54-0.91)	0.52 (0.33-0.82)

**Table 6:** Summary of community-based studies including injectable antibiotics for case management of neonatal sepsis (observational, quasi experimental, and RCT)

Ref and year	Country	Setting	Study design	Therapy given	Other interventions in package	Coverage of antibiotic case mx (% of those who need it)	Intervention group (N/D)	Control group (N/D)	Effect size RR (95% CI)
**Bang 1999**[9]	India	Rural	Non-randomized concurrent control study	Gentamicin IM and cotrimoxazole	Comprehensive perinatal care package including trained TBAs, VHWs undertaking >6 home visits, targeting of small babies for extra support, comm. mobilization for healthy home behaviors etc.	Years 1996-97 85% 685/804 Years 1997-98 93% 913/979	54/1783*	113/2048*	0.56 (0.41-0.77)
**Bhandari 1996**[13]	India	Periurban/ urban	Observational	Cephalexin PO and amikacin IM	None	N/A	124/2007 Age group =1-2 mths	None	No effect size can be calculated CFR= 3.3%
**Baqui 2008**[12]	Bangladesh	Rural	Cluster randomized trial	Procaine penicillin and gentamicin	Birth and newborn-care preparedness postnatal home visits for newborns assessment on 1,3,7 days of birth. Referral when needed	41% estimated from adequacy surveys	82/2812	125/2872	0.66 (0.47-0.93)
**Baqui 2009**[11]	Bangladesh	Rural	Observational **	Procaine penicillin and gentamicin	Referral for very severe disease or possible very severe disease with multiple signs, by CHWs to government subdistrict hospitals. If the family was unable to comply with referral, the CHWs treated local skin and umbilical cord infections with gentian violet and made follow up visits to reassess the infant.	**N/A**	9/204	24/112	0.22 (0.07-0.71) CFR=4.4%

* Combined data from years 2 and 3 of trial i.e. 1996-1997 and 1997-1998.

**Observational data on individual infants evaluated during the cluster randomized trial by Baqui et al. Control group is families unable to comply with referral and were not offered treatment with injectable antibiotics at home.

### Evidence for effectiveness of oral antibiotic therapy alone

**Figure 4:**
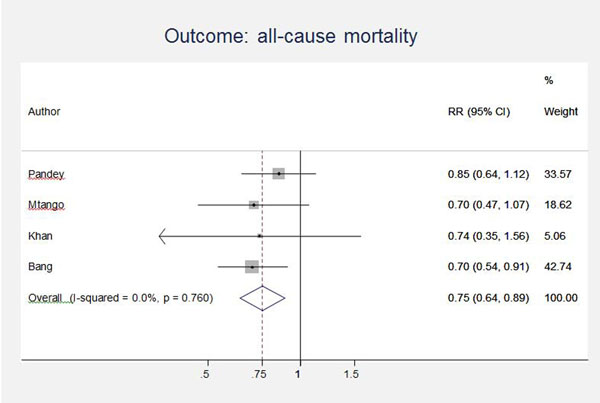
Meta-analysis of observational studies comparing oral antibiotics versus none in the community setting for babies: All cause mortality. Legend: Heterogeneity chi-squared = 1.17 (d.f. = 3) p = 0.760 I-squared (variation in RR attributable to heterogeneity) = 0.0% Test of RR=1 : z= 3.32 p = 0.001

**Figure 5:**
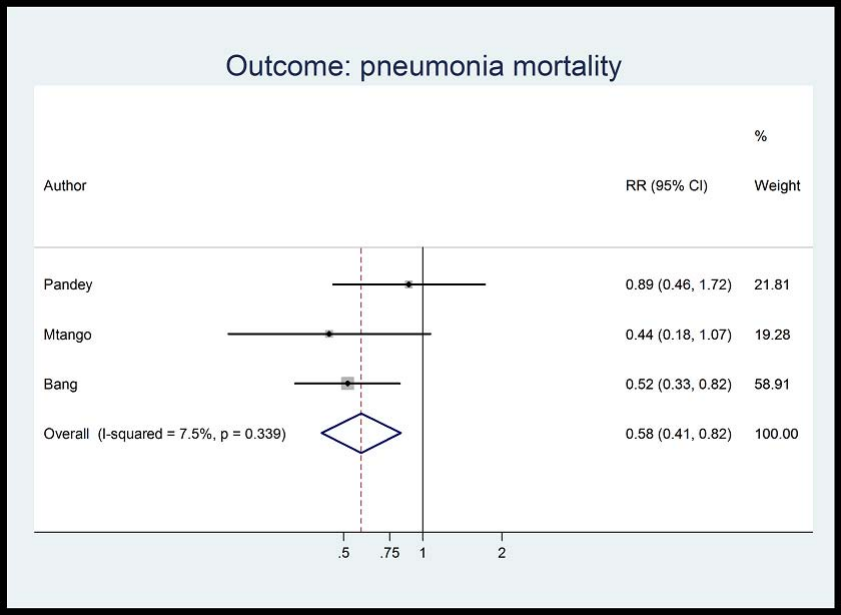
Meta-analysis of observational studies comparing oral antibiotics versus none in the community setting for babies: Pneumonia mortality. Legend: Heterogeneity chi-squared = 2.16 (d.f. = 2) p = 0.339 I-squared (variation in RR attributable to heterogeneity) = 7.5% Test of RR=1: z= 3.06 p = 0.002

**Figure 6:**
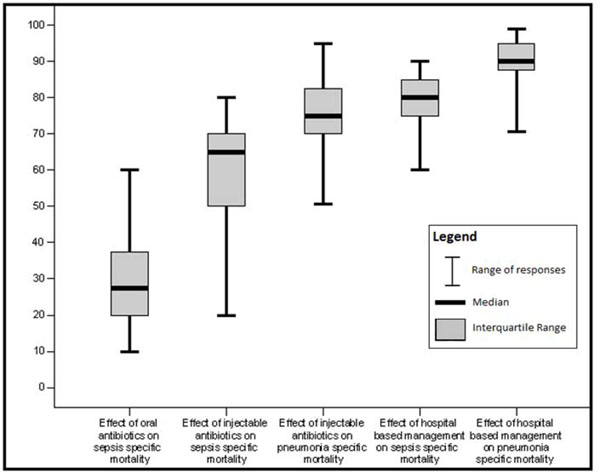
Box plot of Delphi expert opinion estimates of reduction in neonatal cause specific mortality due to pneumonia and sepsis/meningitis

Unpublished neonatal data were obtained from the principal investigators of the four studies identified and a new meta-analysis was done to update that of Sazawal et al[17]. We performed meta analyses for two outcomes: oral antibiotics were associated with reductions in both all-cause mortality (4 studies [10,14-16]: RR 0.75 95% CI 0.64- 0.89) (Figure 4) and pneumonia-specific mortality (3 studies [10,15,16]: RR 0.58 95% CI 0.41- 0.82) (Figure 5). Limitations included non-randomization, estimation of intervention coverage as precise coverage estimates were not available;and variability between studies of the intensity of co-interventions. We found no studies of the effect of oral antibiotics on sepsis-specific mortality. The Delphi consensus (median) was for a 28% reduction in sepsis-specific mortality with an interquartile range of 20% to 36.25% (Figure 6).

### Evidence for effectiveness of injectable antibiotic therapy (±oral antibiotics)

Three studies reported in four papers, were identified (Table 6) [9,11-13]. One, an RCT[12] evaluated the impact of a perinatal care package which included the administration of injectable antibiotics in domiciliary settings in situations where referral to hospital was not possible. This trial reported a reduction in all-cause neonatal mortality of 34% (RR=0.66, 95%CI 0.47-0.93). A second paper from the same study reported that the CFR for neonates who were evaluated and actually treated with injectable antibiotics was 4.4% [11]. A non-randomized, concurrently controlled study [9] also evaluated the impact of a home-based neonatal care package in which septic neonates were treated with injectable antibiotics when referral to hospital was not possible. The overall mortality reduction in the intervention arm of the trial was calculated to be 44% (RR=0.56 95% CI 0.41-0.77). A third, uncontrolled study [13] based in a primary care clinic reported a CFR of 3.3% among septic children treated with injectable antibiotics.

In both of the community-based studies [9,12] injectable antibiotics were only one component of comprehensive community-based newborn care packages, and therefore the effectiveness of injectable antibiotics alone in the community cannot be reliably estimated. The Delphi consensus for the effect of injectable antibiotics was for a 65% reduction (interquartile range of 50-70%) in sepsis-specific mortality and 75% reduction (interquartile range of 70-81.25%) in pneumonia-specific mortality in community-based settings (Figure 6).

### Evidence for effectiveness of inpatient hospital case management

We found no trials assessing the impact of hospital-based case management and the observational studies of hospital management showed wide variation in effect. Searches conducted for studies reporting CFRs in neonates with pneumonia in health facilities revealed very few data. Two studies were identified with author-defined neonatal pneumonia; both were from low income, non-industrialised settings and reported CFRs of 14.4% [72] and 30.8% [71].

**Figure 7:**
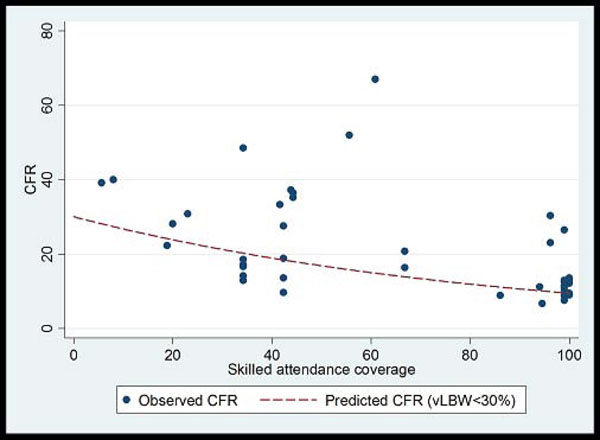
Plot of neonatal sepsis CFR versus percent skilled delivery as a marker of access to facility care. Model fitted: outcome = log(CFR) Covariates = Skilled attendant coverage and % babies vLBW Fitted line is predicted CFR for settings with % VLBW<30%. Predicted CFR at 0% skilled attendance is 30%. Predicted CFR at 100% skilled attendance is 9.5%. % reduction = 68.5% Coefficient skilled attendance is 0.12 on the log scale (95% CI -0.02 to -0.007); i.e. for each 1% increase in skilled attendance rate CFR is reduced by 1.1% (95% CI: 0.7% to 1.6%)

CFRs for neonatal sepsis, adjusted for the proportion of very low birth weight babies in the study, were plotted against national percentage skilled delivery, as a proxy for access to hospital-based case management of neonatal sepsis. In countries with a high proportion of births attended by skilled attendants, the predicted CFR for sepsis was 9.5%, whereas in countries with a low proportion (<30%) skilled birth attendance, the predicted CFR for sepsis with hospital care is 20-30% (Figure 7). A 68% reduction in the CFR for neonatal sepsis is predicted as one moves from 0% to100% skilled birth attendance. This reduction is likely to under estimate the effect of hospital-based case management since skilled birth attendance is likely to be a poor surrogate for effective facility case management of neonatal infections, but was used in the absence of coverage data for case management

Although the quality of evidence is low according to GRADE criteria, the recommendation for case management of neonatal infections is strong, and this is standard practice globally. Table 7 provides a summary of the effect of case management on neonatal sepsis and pneumonia cause specific mortality, and GRADE of the estimate. Therefore the Delphi process was used to provide estimates for the effect of hospital care. The Delphi consensus was for a 80% reduction in sepsis-specific mortality (interquartile range 75% to 85%), and a 90% reduction in pneumonia-specific mortality (interquartile range 88.75% to 95%) (Figure 6).

**Table 7:** Effect of case management on neonatal sepsis and pneumonia cause specific mortality, and GRADE of the estimate

Effect on neonatal deaths due to pneumonia	
***Cause specific effect and 95% CI/ interquartile range:***	
Oral therapy	42% (18-59%,95% CI)
Injection therapy	75% (70-81% interquartile range on Delphi)
Hospital-based case management	90% (89-95% interquartile range on Delphi)

***Quality of input evidence:***	
For oral therapy, moderate (3 low quality non-randomized concurrent control studies)	
For the effects of injection therapy and full case management, the level of evidence is very low (based on Delphi).	

***Proximity of the data to cause specific mortality effect:***	
Moderate for oral therapy as several low quality but consistent studies; however, lack of consistency in cause-of-death definitions	
Very low quality for injection therapy and full case management as these results are based on Delphi	

***Limitations of the evidence:***	
Interpretation of the data is limited by concurrent interventions particularly for studies with injection case management	

***Possible adverse effects:***	
Data not reviewed	

**Effect on neonatal deaths due to sepsis and meningitis**	

***Cause specific effect and interquartile range:***	
Oral therapy	28% (20-36.25% interquartile range on Delphi)
Injection therapy	65% (50-70% interquartile range on Delphi)
Hospital-based management	80% (75-85% interquartile range on Delphi)

***Quality of input evidence:***	
Very low (based on Delphi)	

***Proximity of the data to cause specific mortality effect:***	
Direct effect estimated by Delphi	

***Limitations of the evidence:***	
Lack of direct evidence on sepsis-specific mortality. Studies have evaluated injectable antibiotics as part of multiple co-intervention peri-natal care packages.	

***Possible adverse effects:***	
Data not reviewed	

## Discussion

Infections including sepsis, meningitis and pneumonia are responsible for almost a million neonatal deaths annually. Neonates are more susceptible to severe infections and the progression of disease is more rapid due to developmental immunodeficiency, resulting in high CFRs. Also, a significant proportion of infections may arise early, after vertical transmission from the mother [73]. Therefore, timely identification and appropriate management with antibiotics is an important strategy to reduce the burden of neonatal mortality due to infections. We have previously reported the evidence from observational and experimental studies in low income countries for community-based management of neonatal infections (pneumonia and sepsis) with oral and injectable antibiotics [74-76]. We have now undertaken a systematic review of available evidence, including from industrialized countries and facility settings, and where the quality of evidence is low we have undertaken a Delphi expert process to estimate the cause-specific mortality effect.

This review of effectiveness of the interventions is shaped in large part by the needs of the LiST model. In that model, increasing coverage of an intervention results in a reduction in deaths due to one or more specific causes or in reduction of a risk factor. Therefore the reviews and the GRADE process used were designed to develop estimates of the effect of an intervention in reducing death due to specific causes. For more details of the review methods, the adapted GRADE approach or the LiST model see related publications [6,7].

To our knowledge, this is the first review providing effectiveness estimates for case management options to reduce neonatal deaths due to neonatal sepsis/meningitis and pneumonia, in both community and facility settings. Theodoratou et al have previously estimated effectiveness of pneumonia case management in children under 5 years but they did not disaggregate neonatal mortality data from later child mortality [77]. The estimated effect of community case management on pneumonia mortality in children under 5 years of age in the analysis by Theodoratou et al is 70% (77). Oral antibiotics in community settings for neonatal pneumonia in our analysis were associated with a 42% reduction in pneumonia-specific mortality and a 25% reduction in all-cause neonatal mortality based on a meta-analysis of available trials. There is no evidence to estimate the effect of oral antibiotics on sepsis-specific mortality, but our Delphi process suggested a 28% reduction. Delphi-derived estimates for the effects of management using injectable antibiotics delivered in home or primary care settings came out at 65% for sepsis-specific mortality and 75% for pneumonia-specific mortality. These estimates are biologically plausible and consistent with published studies [9,12] which reported reductions in all-cause neonatal mortality (sepsis plus other causes) of 34% and 44% respectively with community-based packages including injectable antibiotics. CFRs reported from observational studies of hospital case management varied widely, from 6.7 to 67%. Our Delphi estimates suggested an 80% mortality reduction in sepsis deaths and a 90% reduction in pneumonia deaths with hospital case management.

There were 4 effectiveness trials assessing the impact of oral antibiotics on pneumonia-specific mortality in the community. Only one of these studies was randomized and the programmatic coverage of the intervention had to be estimated as coverage data were not routinely assessed or reported. The selection and intensity of co-interventions was not uniform between the studies. An additional limitation was the lack of clearly defined cause-of-death definitions by the authors. However, the effect sizes were remarkably consistent with each other, and therefore the evidence level was upgraded to moderate.

GRADE guidelines rank the evidence relating to the effect of injectable antibiotics on sepsis-specific mortality as low quality. The 3 studies identified were not uniform with respect to study designs; one was an effectiveness RCT, one was a non-randomized concurrent trial and the third was an observational study describing the experience from primary care clinic without a control group. Both the RCT [12] and the non-randomized concurrent trial[9], involved concurrent co-interventions alongside the administration of injectable antibiotics. This made it impossible to assess the impact of injectable antibiotics alone on sepsis mortality. Neither study reported the change in the sepsis-specific mortality rate in the intervention arm compared to control arm, and reported the impact on all-cause neonatal mortality only. The absence of randomization in one of the trials is a further limitation [9]. The main limitation to the observational study in a primary care clinic [13] was the absence of a control arm in the study.

We identified no controlled trials assessing the effect of hospital-based case management of neonatal infections. Such studies would be difficult or impossible to implement in an ethical fashion. Thus studies were limited to reporting CFRs for neonatal sepsis and meningitis. The studies were from varied settings, from both industrialized and low income countries, and reported widely varying CFRs. Only 2 of these observational studies reported CFRs for pneumonia. One of these studies reported a very high CFR for pneumonia due, we believe, to the high proportion of LBW babies in the sample (60%).

We found some moderate quality evidence for intervention packages including antibiotics in community settings but ironically data are most lacking at facility level, and district hospital level is a critical gap [78]. Unlike the LiST review on neonatal resuscitation which identified several before-after studies of facility based neonatal resuscitation reporting mortality data, we were unable to find similar before-after studies on the effect of hospital-based case management of sepsis/meningitis/pneumonia. An understandable reason for this might be the ethical constraints precluding such studies. However, historical reviews from the pre-antibiotic era provide an insight into the CFR associated with untreated sepsis in facility settings. The best available evidence comes from the series of papers from Yale Medical Center reporting time trends for neonatal sepsis. These data show that in the 1920s and 1930s the CFR for blood culture confirmed sepsis stood at 90% [79,80]. With the introduction of antibiotics, the CFR decreased to 45% by 1965 [81], and with the subsequent introduction of intensive care units and advanced life support it came down to 16% by 1988[82], and 3% by 2003[83]. Such data highlight the effectiveness of hospital-based management in preventing neonatal mortality from sepsis.

## Conclusion

As evident from our results, even oral or injectable antibiotics alone are highly effective in reducing deaths from neonatal sepsis or pneumonia. These interventions hold great potential to reduce the 1 million neonatal deaths each year. If substantial reduction in neonatal mortality is desired, both, community and facility-based interventions are required, linked by functioning referral systems, giving the potential to prevent hundreds of thousands of avoidable newborn deaths every year.

## List of abbreviations used

CFR: Case Fatality Rate; CHERG: Child Health Epidemiology Reference Group; IMCI: Integrated Management of Childhood Illnesses; LiST: Lives Saved Tool; RCT: Randomized Controlled Trial.

## Competing interests

The authors declare that they have no competing interests.

## Authors’ contributions

AZ and JL planned the review, SS and AZ undertook the searches and abstraction with input from JL and HG. SC undertook the meta-analyses. RB provided unpublished data from a previous investigator working group. JL, AZ and ACCL planned the Delphi. All authors contributed to the manuscript.

## Funding

This work was supported in part by a grant to the US Fund for UNICEF for Child Health Epidemiology Reference Group from the Bill &Melinda Gates Foundation (grant 43386) to “Promote evidence-based decision making in designing maternal, neonatal and child health interventions in low- and middle-income countries”, and by a grant to Save The Children USA from the Bill & Melinda Gates Foundation (Grant 50124) for "Saving Newborn Lives".

## Supplementary Material

Additional file 1 Study identifiers and context Click here for file
